# Enhancing Chronic-Disease Education through Integrated Medical and Social Care: Exploring the Beneficial Role of a Community Teaching Kitchen in Oregon

**DOI:** 10.3390/nu15204368

**Published:** 2023-10-14

**Authors:** Jacob P. Tanumihardjo, Heidi Davis, Mengqi Zhu, Helen On, Kayla K. Guillory, Jill Christensen

**Affiliations:** 1 Section of General Internal Medicine, University of Chicago, Chicago, IL 60637, USA; jtanumihardjo@bsd.uchicago.edu (J.P.T.);; 2 Community Teaching Kitchen, Providence Milwaukie Hospital, Providence Health & Services, Milwaukie, OR 97222, USA; kayla.guillory@providence.org (K.K.G.);; 3 Population Health Division, Providence Health & Services, Portland, OR 97213, USA; nhan.on@providence.org

**Keywords:** teaching kitchens, food insecurity, chronic disease, social determinants of health, nutrition

## Abstract

Teaching kitchens (TKs) are rapidly being utilized as models to integrate culinary education and chronic-disease education into healthcare settings. Our observational study details the structure and organizational processes (e.g., referral, services, medical and social care integration) of the Community TK at Providence Milwaukie Hospital in Portland, OR. We utilize electronic medical-record data from engaged TK participants (*n* = 3077) to evaluate between the association of engagement and clinical outcomes (e.g., HbA1c, blood pressure, weight and cholesterol). Mean baseline HbA1c of Highly Engaged TK patients with diabetes (*n* = 88) reduced from 9.8% to 8.6% at 6 months (*p* < 0.0001) and sustained significant reductions at 12, 18, 24, 30, and 36 months (*p* < 0.05). Highly Engaged patients with hypertension (*n* = 152) had significant, sustained reductions in blood pressure (*p* < 0.0001). Engaged patients in the same high-risk groups also had significant improvements in HbA1c and blood pressure. Both engagement subgroups had moderate improvements in weight change and cholesterol. This study shows promising associations of TK services that promote chronic-disease self-management with improved clinical outcomes among higher risk patients (e.g., high blood pressure, high HbA1c, high low-density lipoprotein) with different medical issues (e.g., diabetes, obesity) and social barriers (e.g., food insecurity).

## 1. Introduction

The prevalence of diabetes and other chronic conditions has reached alarming levels worldwide, posing significant challenges for healthcare systems and individuals alike [1,2,3]. Chronic conditions such as diabetes, cardiovascular disease, and chronic kidney disease represent some of the leading causes of morbidity and mortality globally [4,5]. While medication and traditional therapies remain essential components of disease management, the importance of diet and lifestyle modifications cannot be overstated [6,7,8]. Inadequate nutritional knowledge, limited access to healthy foods, and dietary choices with low nutritional value contribute to worsening chronic conditions, leading to increased morbidity, mortality, and healthcare costs.

Culinary medicine, an interdisciplinary field that merges the art of cooking and nutritional science, offers a novel approach to empower individuals in their pursuit of better health outcomes. By combining culinary skills with evidence-based dietary recommendations, healthcare providers can equip patients with the practical knowledge and tools necessary to make informed food choices. Cooking techniques, ingredient selection, and personalized meal planning are tailored to individual dietary needs, empowering patients to take an active role in managing their conditions and promoting overall well-being. However, the implementation of culinary medicine programs should not neglect the significant challenges posed by food insecurity. Food insecurity, defined as a lack of consistent access to enough food for every person in a household to live an active, healthy life, affects a substantial portion of individuals with chronic conditions [9,10]. This issue encompasses both urban and rural communities, crossing physical boundaries and necessitating multi-faceted policy decisions to address barriers for the communities impacted [11,12]. Food insecurity not only compromises the nutritional status of individuals but also exacerbates health disparities and increases the burden of chronic diseases [13,14]. Addressing food insecurity is essential for optimizing the impact of culinary medicine interventions and ensuring equitable healthcare access for all [15]. Furthermore, the prevalence of food insecurity among individuals with chronic conditions adds an additional layer of complexity to overall care, especially among those who face poverty and other structural health determinants [16,17]. Addressing food insecurity not only ensures access to nutritious food but also empowers individuals to make healthier choices [18].

One innovative model that effectively integrates culinary medicine and chronic-disease education is the establishment of teaching kitchens [19,20,21,22,23]. These kitchens serve as a transformative space where individuals can learn, practice, and share culinary skills and nutrition knowledge. Teaching kitchens (TKs) not only provide a supportive environment for individuals to develop practical cooking skills but also foster a sense of community, empowerment, and shared experience. By addressing both the nutritional and social aspects of chronic-disease care, these kitchens offer a holistic approach that promotes sustained behavior change and improved health outcomes. In this observational study, we aim to describe the structure and organizational processes (e.g., referral, services, medical and social care integration) of a community-based TK integrated within a medical setting that incorporates food-insecurity screening and food-provision support within its culinary medicine programs and chronic-disease education. We will examine clinical outcomes such as HbA1c, blood pressure, weight, and cholesterol amongst patients engaged with Providence Community TK services and if engagement with TK services was associated with improved, or stabilized, outcomes. By shedding light on this innovative approach, we hope to inspire healthcare providers, policymakers, and community leaders to embrace culinary medicine and food insecurity support as integral components of comprehensive chronic-disease management, treatment, and prevention. Together, culinary medicine and TKs have the potential to revolutionize the landscape of diabetes and chronic-disease care, promoting long-lasting lifestyle changes, fostering community resilience, and ultimately improving health outcomes for individuals facing complex health challenges.

## 2. Materials and Methods

### 2.1. Overview

This study included the active adult population within the community-based TK at Providence Milwaukie Hospital from January 2016 to December 2022. Electronic medical record (EMR) data were utilized to evaluate the effects of Providence’s community-based TK. This study was approved by the Institutional Review Boards at the University of Chicago and Providence St. Joseph Health.

### 2.2. Setting

Providence Milwaukie Hospital is part of Providence St. Joseph Health, an integrated health-delivery system with eight hospitals and 100+ primary care and specialty clinics in Oregon, USA. The Providence Community TK is situated at Providence Milwaukie Hospital and is focused on collaborating with the community to provide preventative health programs and services. The Providence Community TK primarily serves individuals who face food insecurity in the Multnomah and Clackamas counties in Oregon, a population identified as medically underserved, people whose income is under or near the federal poverty threshold, and with significant chronic disease [24].

### 2.3. Teaching Kitchen Referrals

In 2016, the Providence Community TK was established with dedicated protocols to refer adult patients to internal services to meet food-insecurity barriers and medical nutrition-therapy needs. During early years of implementation (2016–2018), patients were referred for services based on the presence of food insecurity and/or diagnosis codes associated with medical nutrition therapy needs (i.e., obesity, uncontrolled type 2 diabetes, hypertension, malnutrition). Recognizing the need to centralize services for patients who had built a trusting relationship with TK providers, the Providence Community TK became certified as a Diabetes Education Center in 2019. This transition enabled a “one-stop-shop” model to more holistically serve the medical and social needs of patients and a growing community of referring healthcare providers. As diabetes education services began to expand in 2019, Providence Community TK referrals became more medically tailored to address chronic-disease complications related to type 2 diabetes. In 2020, faced with difficulties from the pandemic, the Providence Community TK began to facilitate virtual services for patients and relied heavily on referrals from providers as well as self-referrals from patients. The pandemic proved to be a time to focus directly on billing/reimbursement for TK services, implementing and expanding patient insurance verification practices, and for diversification of revenue streams. In 2021 and 2022, TK services were available to community members via medical, self, or community-based referrals, and staff were trained to assess patients’ needs and initiate referrals based on clinical diagnoses, educational needs, or social factors [25].

### 2.4. Providence Community Teaching Kitchen Design and Evolution

The Providence Community TK has five components: chronic-disease self-management education, culinary nutrition education, patient navigation, a medical referral-based food pantry (Family Market), and an immersive environment to support physician resident, dietetic intern, and behavioral health therapist training [25]. In 2016, the Providence Community TK was established with a patient navigator (1.0 FTE), a registered dietitian (1.0 FTE), and a physician champion (Table 1). With additional support from the Providence Milwaukie Hospital Foundation, the Providence Community TK expanded to two patient navigators (1.5 FTE) and two registered dietitians (2.0 FTE) in 2017. As the program continued to mature and billing for services improved in 2018, a dedicated program manager was required (1.0 FTE) to oversee day-to-day operations of the TK. In addition, the Providence Community TK began expanding available services to include a dedicated cooking instructor that supported class instruction and video development. In 2020, the Providence Community TK expanded to include a videographer to support media development, and in 2021 that role continued to expand in scope (0.75 FTE). In 2022, the Providence Community TK received health-system funding, dedicated to promoting health equity, to expand an onsite gardening program, and a dedicated Gardening Coordinator (0.5 FTE) was added to the team.

### 2.5. Addressing Unmet Social Needs

Providence St. Joseph Health in Oregon developed a comprehensive intervention designed to simultaneously address the clinical challenges of chronic conditions and the social-need challenges that exacerbate medical care for underserved populations [26]. In addition to this multi-year effort, Providence St. Joseph Health also integrated social-needs screening and referral activities into clinical settings with dedicated support from a program, co-developed with a local social-service agency, called the Community Resource Desk [26,27]. The Providence Community TK and it’s staff were integrated into these interventions. The Providence TK has a dedicated on-site food pantry, the Family Market, that uses a grocery store shopping model to tailor food access according to individual needs [25]. Providence hospital and clinical staff regularly screen patients for social needs (e.g., food insecurity, utilities, housing, transportation) and refer them to TK services (e.g., food provision, medical nutritional therapy, individual counseling, and group culinary classes) or to the Community Resource Desk to support patients who screen positive for other unmet needs.

### 2.6. Study Population

This study included adult patients (18 years or older) with at least one confirmed encounter with the Providence Community TK between 1 January 2016 and 31 December 2022. Providence staff classify encounters within the EMR based on various factors including setting appointments, mode of appointment, and follow-up (e.g., phone call, patient portal message). To define confirmed intervention encounters, the research team focused on encounters with designations that describe office, telephone, or virtual visits.

To account for level of engagement, intervention subgroups were assigned to evaluate trends in clinical outcomes among those engaged with the Providence Community TK. Engaged patients were defined as those with one or two confirmed encounters with the Providence Community TK during the study. Highly Engaged patients were defined as those with three or more confirmed encounters with the Providence Community TK during the study. The Providence Community TK began in 2016 and baseline start dates were assigned to patients within the Engaged and Highly Engaged subgroups based on their first confirmed encounter with any Providence Community TK provider after 1 January 2016.

### 2.7. Data Measures and Subpopulation Analyses

Patient demographics, outpatient encounter data, clinical lab values (e.g., HbA1c, low-density lipoprotein (LDL), systolic blood pressure (SBP), diastolic blood pressure (DBP), weight), patient problem lists (i.e., ICD-10 diagnosis codes), and social needs screening results were extracted from the EMR by Providence research staff. The Providence Community TK serves patients based on both medical needs (e.g., hypertension, uncontrolled type 2 diabetes, malnutrition) and social needs (e.g., food insecurity, difficulty affording healthy foods). Due to this variability, clinical outcomes were evaluated based on clinical risk factors and longitudinal data was composed to understand individual clinical outcomes in the context of patient engagement. Blood sugar control measures were evaluated among patients with a baseline HbA1c greater than, or equal to, 8% [28]. Blood pressure measures were evaluated for patients with a baseline SBP reading greater than, or equal to, 130 mmHg and a baseline DBP reading greater than, or equal to, 80 mmHg [29]. Cholesterol measures were evaluated for patients with a baseline LDL greater than, or equal to, 100 mg/dL [30]. These clinical outcome cutoffs align with Providence’s clinical-quality-improvement metrics for patients with diabetes, hypertension, and hyperlipidemia. Weight was evaluated across the entire study population and is represented in overall percent of weight change to align with weight-management programs [31]. HbA1c, blood pressure, and weight change were evaluated at 6-month intervals while cholesterol was evaluated at 12-month intervals to align with annual lipid panel screenings.

### 2.8. Statistical Analyses

All statistical analyses were performed using R Studio (R version 4.2.0). We compared characteristics among groups and conducted self-comparisons in clinical outcomes over time per group. Chi-square tests and Fisher’s exact tests were conducted to compare binary and multinomial characteristics (e.g., sex, race and ethnicity, co-morbidity prevalence, social needs, age group) between groups. A Wilcoxon rank sum test was used to compare continuous age. We also conducted linear mixed models and generalized linear mixed models within each group to evaluate any time-trend effect and to compare each subsequent time point to baseline for continuous clinical outcomes (e.g., HbA1c, blood pressure, LDL, weight), using Tukey’s method to adjust for multiple comparisons. Significance testing was conducted within groups due to significant differences between study groups in demographic characteristics, social needs, and disease burden, and the adopted level of significance was *p* < 0.05.

## 3. Results

### 3.1. Population Description (Table 2)

We identified 3077 patients in the EMR with confirmed encounters with the Providence Community TK. Overall, patients were predominantly White (79.8%) and identified as female (66%). Patients varied in age groups as well as insurance coverage (Table 2). There were significant differences between Engaged and Highly Engaged patients in insurance type, race and ethnicity, age, and social needs. Engaged patients were younger (*p* = 0.013), more likely to be non-White (*p* = 0.009), and more often covered by Commercial/Other insurance types when compared to Highly Engaged patients (*p* < 0.001). Highly Engaged patients had a higher prevalence of each social need (e.g., food insecurity, housing, transportation, utilities) (*p* < 0.01), were more likely obese (*p* = 0.049) or had depressive disorders (*p* = 0.005), and were more likely to covered by Medicare, Medicaid, or Dual Eligible (*p* < 0.001).

**Table 2:** Demographics of patients engaged with the Providence Community Teaching Kitchen.

	Overall, N = 3077	Highly Engaged, N = 959	Engaged, N = 2118	*p*-Value ^1^
Insurance Type, N (%)				<0.001
Commercial/Other	1049 (35.2)	249 (26.6)	800 (39.1)	
Medicare	1033 (34.7)	375 (39.1)	658 (32.2)	
Medicaid	828 (27.8)	283 (30.4)	545 (26.7)	
Dual Eligible	69 (2.3)	28 (3.0)	41 (2.0)	
Missing	98	24	74	
Gender/Sex, N (%)				0.2
Female	2038 (66.2)	656 (68.4)	1382 (65.2)	
Male	1037 (33.7)	302 (31.5)	735 (34.7)	
Other	2 (0.1)	1 (0.1)	1 (0.1)	
Mean Age (SD)	51.4 (16.2)	52.3 (15.6)	50.9 (16.4)	0.013
Age Group, N (%)				0.075
18–39	812 (26.3)	224 (23.4)	588 (27.8)	
40–49	545 (17.7)	164 (17.1)	381 (18.0)	
50–59	645 (21.0)	216 (22.5)	429 (20.3)	
60–64	355 (11.5)	118 (12.3)	237 (11.2)	
65+	720 (23.4)	237 (24.7)	483 (22.8)	
Race/Ethnicity, N (%)				0.009
White, Non-Hispanic	2357 (79.8)	743 (80.8)	1614 (79.4)	
Other Race, Non-Hispanic	173 (5.9)	63 (6.8)	110 (5.4)	
Asian, Native Hawaiian or Other Pacific Islander, Non-Hispanic	102 (3.5)	23 (2.5)	79 (3.9)	
Black or African American, Non-Hispanic	78 (2.6)	31 (3.4)	47 (2.3)	
Hispanic or Latino, of any race	244 (8.3)	60 (6.5)	184 (9.0)	
Missing	123	39	84	
Social Needs, N (%)				
Food Insecurity	608 (19.8)	315 (32.8)	293 (13.8)	<0.0001
Housing	86 (2.8)	45 (4.7)	41 (1.9)	<0.0001
Transportation	112 (3.6)	55 (5.7)	57 (2.7)	<0.0001
Utilities	78 (2.5)	36 (3.8)	42 (2.0)	0.006
Comorbidities, N (%)				
Obesity	436 (14.2)	154 (16.1)	282 (13.3)	0.049
Diabetes mellitus with/without complication	401 (13.0)	120 (12.5)	281 (13.3)	0.6
Disorders of lipid metabolism	368 (12.0)	112 (11.7)	256 (12.1)	0.79
Hypertension	365 (11.9)	117 (12.2)	248 (11.7)	0.74
Depressive disorders	281 (9.1)	109 (11.4)	172 (8.1)	0.005
Osteoarthritis	222 (7.2)	80 (8.3)	142 (6.7)	0.12
Heart failure	145 (4.7)	45 (4.7)	100 (4.7)	1
Coronary atherosclerosis and other heart disease	141 (4.6)	46 (4.8)	95 (4.5)	0.77
Chronic kidney disease	130 (4.2)	44 (4.6)	86 (4.1)	0.56
3 or more chronic conditions	322 (10.5)	112 (11.7)	210 (9.9)	0.16

^1^ Pearson’s Chi-squared test; Fisher’s exact test; Wilcoxon rank sum test.

### 3.2. Clinical Measures

**Figure 1:**
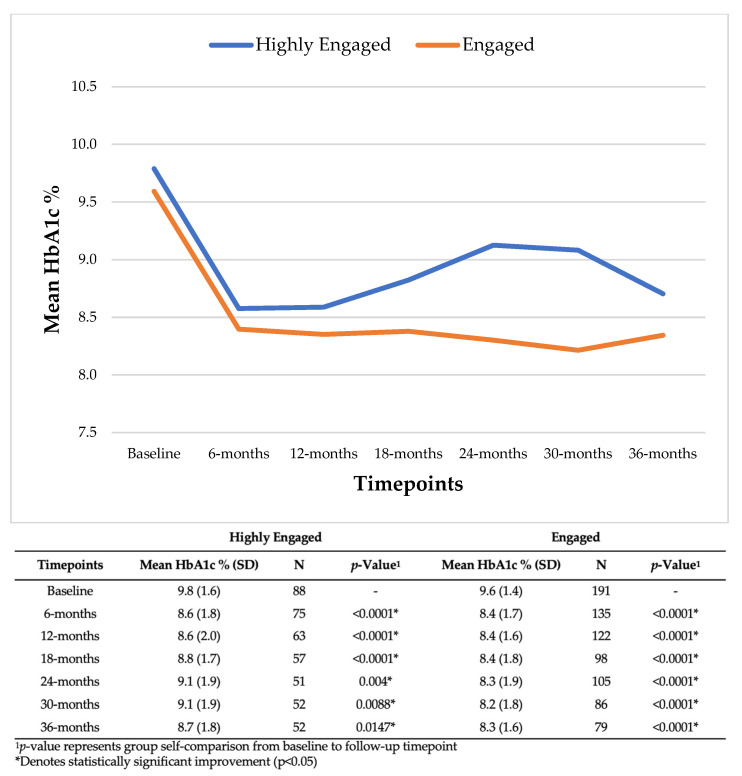
Mean HbA1c among high-risk patients with diabetes (HbA1c >8%) at the Providence Community Teaching Kitchen.

High-risk patients with diabetes (baseline HbA1c > 8%) at the Providence Community TK reduced their HbA1c by 1.2% from baseline to 6 months for both the Highly Engaged [9.8% (SD: 1.6) to 8.6% (SD: 1.8), *p* < 0.0001] and Engaged groups [9.6% (SD: 1.4) to 8.4% (SD: 1.7), *p* < 0.0001], and both groups sustained significant reductions at 12, 18, 24, 30, and 36 months (Figure 1).

Patients with hypertension (baseline SBP > 130 mmHg and DBP > 80 mmHg) at the Providence Community TK, in both study groups, had significantly reduced SBP and DBP at each subsequent timepoint (e.g., 6-, 12-, 18-, 24-, 30-, 36 months) from baseline values (Table 3). Highly Engaged patients with hypertension had significant reductions of baseline SBP and DBP, 141.4 mmHg (SD: 10.2) and 86.8 mmHg (SD: 6.0), respectively, to 134.2 mmHg (SD: 15.7) and 81.2 mmHg (SD: 8.8), respectively, after 6 months (*p* < 0.0001). Among Engaged patients with hypertension, baseline SBP and DBP were also significantly reduced from 142.4 mmHg (SD: 11.0) and 87.5 mmHg (SD: 6.2), respectively, to 136.2 mmHg (SD: 14.0) and 82.2 mmHg (SD: 9.1), respectively, after 6 months (*p* < 0.0001). Significant reductions in both the Highly Engaged and Engaged groups continued even after 36 months (*p* < 0.0001).

Among all Highly Engaged patients at the Providence Community TK with weight data, significant weight change from baseline, as measured by percentage, occurred at 6 months [−0.83% (SD: 5.30), *p* = 0.0012], 12 months [−0.90% (SD: 6.37), *p* = 0.0001], 18 months [−1.03% (SD: 7.34), *p* < 0.0001], 24 months [−1.22% (SD: 8.30), *p* < 0.0001], 30 months [−1.46% (SD: 8.88), *p* < 0.0001], and 36 months [−1.25% (SD:9.62), *p* < 0.0001] (Table 4). Among all Engaged patients with weight data, significant weight change from baseline, as measured by percentage, occurred at 6 months [−0.36% (SD: 5.55), *p* = 0.0431], 18 months [−0.24% (SD: 8.00), *p* = 0.0183], 24 months [−0.30% (SD: 8.77), *p* = 0.0021], and 30 months [−0.35% (SD: 9.09), *p* = 0.0003] (Table 4). There were trends in weight change among Engaged patients at 12 months and 36 months (*p* < 0.10).

Patients with abnormal cholesterol (baseline LDL > 100 mg/dL) at the Providence Community TK, in both study groups, had significantly reduced LDL at each subsequent timepoint (e.g., 12-, 24-, 36 months) from baseline values (Table 5). Highly Engaged patients with abnormal cholesterol had significant reductions in baseline LDL from 133.5 mg/dL (SD: 28.5) to 120.2 mg/dL (SD: 34.6) after 12 months (*p* < 0.0001). Among Engaged patients with abnormal cholesterol, baseline LDL was significantly reduced from 134.8 mg/dL (SD: 25.3) at baseline to 121.7 mg/dL (SD: 29.0) after 12 months (*p* < 0.0001).

## 4. Discussion

Other TK programs have shown improvements in clinical, behavioral, and self-reported outcomes in focused populations (e.g., medical student cohorts, breast cancer survivors, patients with type 2 diabetes, perimenopausal women) [32,33,34,35]. Our observational study is one of the first manuscripts to evaluate the multi-functional role of a community-based TK in improving clinical outcomes among a heterogeneous patient population with variable primary diagnoses and clinical outcomes of interest. Among both engagement subgroups, we found significant improvements in blood sugar control, blood pressure maintenance, cholesterol management, and weight change. Notably, these improvements in clinical outcomes are in the Highly Engaged subgroup with higher incidences of unmet social needs (e.g., food insecurity, housing, transportation, utilities) and certain medical conditions (e.g., obesity, depressive disorders) when compared to the Engaged subgroup. Clinical improvements amongst a population that faces difficult medical and social needs are crucial for advancing health equity as these patients face significant health disparities [14,36,37]. For high-risk patients with diabetes, both engagement subgroups had mean reductions in HbA1c at each follow-up timepoint including 1.2% reduction at 12 months and greater than 1% reduction at 36 months. A 1% reduction in HbA1c has been associated with reduced macrovascular and microvascular complications as well as reduced healthcare costs [38,39]. For high-risk patients with hypertension, both engagement subgroups had mean SBP and DBP reductions of greater than 6 mmHg at each follow-up timepoint. Mean reductions in SBP (e.g., 5 mmHg increments) and DBP (e.g., 2 mmHg increments) have been associated with reduced stroke risk among patients with diabetes [40]. HbA1c and blood pressure control are critical outcomes for population health management strategies at Providence St. Joseph Health and the Providence Community TK has shown to be an effective health-promotion program in achieving these goals among high-risk patients. Patients across both engagement groups also had reductions in both weight and LDL. In both cases though, reductions did not meet increments (e.g., 5% weight loss, ≥50% LDL reduction) that could be clinically relevant [41,42]. Although our study does not account for other health interventions, including prescription medication. The association of engagement in TK services with sustained positive health outcomes suggests that this increased level of support and education can be a key factor in chronic-disease management, especially for high-risk patients with unmet social needs.

Based on our understanding of current TK models, other TKs often do not incorporate an on-site food-assistance program that supports food-insecurity needs of both patients and their families. In addition, screening for food insecurity and other unmet social needs is not a universal practice among TKs. Healthcare workflows that address unmet social needs and alleviate social and structural drivers of health are considered among the best practices of primary care [43,44]. The combination of screening for unmet needs with readily available food resources is a unique quality of the Providence Community TK and provides healthcare providers access to a community-based program that can alleviate both chronic-disease education needs as well as food-access barriers [18].

This study has several limitations. We were unable to integrate information on food-insecurity support based on food-provision assistance due to how referral and usage data are stored. For example, the Family Market program data are not integrated into the Providence EMR. Future studies and existing TKs should consider pathways that ensure social-needs referral and usage data are accessible and embedded into EMR workflows to allow for additional research into the role of TKs in alleviating food-insecurity barriers for patients. We evaluated the entire population served by the Providence Community TK and our evaluation reflects a heterogenous population of patients that face different medical conditions (e.g., obesity, diabetes, hypertension, malnutrition), and presenting clinical outcomes universally across the population would not be representative of clinical goals for individual patients. To account for this issue, we evaluated each subgroup based on baseline clinical outcomes that align with quality improvement and clinical performance metrics (e.g., HbA1c > 8%, SBP > 130 mmHg/DBP > 80 mmHg, LDL > 100 mg/dL). It is important to note that we were not able to evaluate statin or other treatment prescriptions within our analyses. Future studies should evaluate subpopulations in greater detail, such as incorporating statin prescription within cholesterol analyses, and should integrate patient-reported outcomes (e.g., satisfaction, food insecurity, activation, empowerment) into their evaluations to better understand patient outcomes. We did not conduct difference-in-difference analyses to evaluate if varied or continuous engagement led to improved outcomes between the subgroups or to a comparison group. Patient subgroups differed significantly in demographic characteristics (e.g., age, race/ethnicity), medical needs (e.g., obesity), and social needs (e.g., insurance coverage, food insecurity), and a comparison of outcomes and would not provide meaningful interpretations. Future studies should consider the effect that insurance type and coverage can have on the accessibility of TK services and potential out-of-pocket costs for individuals who may benefit from TK support. Lastly, we did not evaluate and isolate the impact of the COVID-19 pandemic on outcomes or engagement. Patients in both subgroups varied in their enrollment timelines and isolating calendar year analyses would have led to a reduction in available data across high-risk clinical subgroups.

## 5. Conclusions

The results of this evaluation show that participating in the Providence Community TK program was associated with significant improvements in clinical outcomes, such as reductions in HbA1c, decreased blood pressure, weight loss, and decreased cholesterol. These clinical improvements align with existing population-health-management efforts to improve federal and state clinical measures in the United States [28,45,46]. The Providence Community TK provides a blueprint for hospital systems, and other clinical settings, that are seeking TK structures and processes that integrate medical and social care models that holistically support chronic-disease education, food-insecurity and nutrition support, and culinary education opportunities for patients.

## Figures and Tables

**Table 1:** Full-time staff equivalents for the Providence CTK.

Year	2016	2017	2018	2019	2020	2021	2022
CTK Staff Member (FTE)	Patient Navigator (1.0 FTE)	Patient Navigator (1.5 FTE)	Patient Navigator (1.5 FTE)	Patient Navigator (1.5 FTE)	Patient Navigator (1.5 FTE)	Patient Navigator (1.5 FTE)	Patient Navigator (2.0 FTE)
	Registered Dietitian (1.0 FTE)	Registered Dietitian (2.0 FTE)	Registered Dietitian (2.0 FTE)	Registered Dietitian (2.0 FTE)	Registered Dietitian (1.5 FTE)	Registered Dietitian (1.5 FTE)	Registered Dietitian (2.5 FTE)
	Physician Champion	Physician Champion	Physician Champion	Physician Champion	Physician Champion	Physician Champion	Physician Champion
			Program Manager (1.0 FTE)	Program Manager (1.0 FTE)	Program Manager (1.0 FTE)	Program Manager (1.0 FTE)	Program Manager (1.0 FTE)
			Cooking Instructors (0.5 FTE)	Cooking Instructors (0.25 FTE)	Cooking Instructors (0.25 FTE)	Cooking Instructors (0.25 FTE)	Cooking Instructors (0.5 FTE)
					Videographer (0.25 FTE)	Videographer (0.75 FTE)	Videographer (0.75 FTE)
							Gardening Coordinator (0.5 FTE)
Total FTE	2.0	3.5	5.0	4.75	4.5	5.0	7.25

**Table 3:** Blood pressure control among hypertensive (SBP > 130/DBP > 80 mmHg) Providence Community Teaching Kitchen patients.

	Highly Engaged				Engaged			
Timepoint	Mean SBP (SD)	Mean DBP (SD)	N	*p*-Value ^1^	Mean SBP (SD)	Mean DBP (SD)	N	*p*-Value ^1^
Baseline	141.4 (10.2)	86.8 (6.0)	152	-	142.4 (11.0)	87.5 (6.2)	338	-
6 months	134.2 (15.7)	81.2 (8.8)	107	<0.0001 *	136.2 (14.0)	82.2 (9.1)	222	<0.0001 *
12 months	134.2 (12.8)	80.6 (8.3)	98	<0.0001 *	135.4 (15.1)	82.5 (9.9)	207	<0.0001 *
18 months	134.1 (14.4)	79.7 (9.5)	84	<0.0001 *	134.3 (14.8)	81.6 (8.7)	178	<0.0001 *
24 months	132.0 (14.0)	78.9 (8.9)	79	<0.0001 *	135.5 (14.7)	81.4 (9.2)	146	<0.0001 *
30 months	131.2 (12.6)	79.3 (9.6)	78	<0.0001 *	134.4 (14.6)	80.7 (8.8)	145	<0.0001 *
36 months	132.2 (12.9)	79.4 (6.9)	66	<0.0001 *	135.0 (16.0)	81.4 (8.9)	126	<0.0001 *

Note: Blood pressure measurement units—mmHg, SBP—Systolic Blood Pressure, DBP—Diastolic Blood Pressure. ^1^ *p*-value represents group self-comparison from baseline to follow-up timepoint. * Denotes statistically significant improvement (*p* < 0.05).

**Table 4:** Percent Weight Change among Providence Community Teaching Kitchen Patients.

	Highly Engaged				Engaged			
Comparisons	Mean Change (%)	SD	N	*p*-Value	Mean Change (%)	SD	N	*p*-Value
Baseline to 6 months	−0.83	5.30	707	0.0012 *	−0.36	5.55	1299	0.0431 *
Baseline to 12 months	−0.90	6.37	606	0.0001 *	−0.27	6.75	1162	0.0842
Baseline to 18 months	−1.03	7.34	509	<0.0001 *	−0.24	8.00	957	0.0183 *
Baseline to 24 months	−1.22	8.30	474	<0.0001 *	−0.30	8.77	909	0.0021 *
Baseline to 30 months	−1.46	8.88	433	<0.0001 *	−0.35	9.09	854	0.0003 *
Baseline to 36 months	−1.25	9.62	390	<0.0001 *	−0.16	9.64	774	0.0774

* Denotes statistically significant improvement (*p* < 0.05).

**Table 5:** Low-density lipoprotein among high-risk patients with abnormal cholesterol (>100 mg/dL) at the Providence Community Teaching Kitchen.

	Highly Engaged			Engaged		
Timepoint	Mean LDL (SD)	N	*p*-Value ^1^	Mean LDL (SD)	N	*p*-Value ^1^
Baseline	133.5 (28.5)	120	-	134.8 (25.3)	300	-
12 months	120.2 (34.6)	46	<0.0001 *	121.7 (29.0)	109	<0.0001 *
24 months	106.1 (32.5)	36	<0.0001 *	129.6 (32.9)	88	<0.0001 *
36 months	112.5 (37.4)	32	<0.0001 *	119.5 (32.2)	78	<0.0001 *

Note: LDL—Low-density lipoprotein. ^1^ *p*-value represents group self-comparison from baseline to follow-up timepoint. * Denotes statistically significant improvement (*p* < 0.05).

## Data Availability

The clinical outcomes and demographic data in the article will not be made available because permission was not obtained from the study participants to share their data publicly. Other data regarding teaching kitchen structure and process can be made available upon request.
